# Will Fly Repellency Using Deltamethrin Reduce Intramammary Infections, Stress and Fatigue Indicators of Dairy Ewes under Intensive Management?

**DOI:** 10.3390/pathogens10020232

**Published:** 2021-02-19

**Authors:** Konstantinos V. Arsenopoulos, Georgios Sioutas, Eleutherios Triantafillou, Athanasios I. Gelasakis, Elias Papadopoulos

**Affiliations:** 1 Laboratory of Parasitology and Parasitic Diseases, School of Veterinary Medicine, Faculty of Health Sciences, Aristotle University of Thessaloniki, 54124 Thessaloniki, Greece; arsenopo@vet.auth.gr (K.V.A.); gsioutas@vet.auth.gr (G.S.); 2 Vet Analyseis, Veterinary Microbiological Laboratory, Karditsis 98 str., 41222 Larissa, Greece; eltriantafil@gmail.com; 3 Laboratory of Anatomy and Physiology of Farm Animals, Department of Animal Science, Agricultural University of Athens (AUA), Iera Odos 75 str., 11855 Athens, Greece; gelasakis@aua.gr

**Keywords:** deltamethrin, dairy ewes, mastitis, fly repellency, somatic cell counts, cortisol, creatine kinase, Greece

## Abstract

Intramammary infections (IMIs) caused by various pathogens may lead to clinical or subclinical mastitis, challenging the health and welfare status of infected animals and decreasing the quantity and quality of the produced milk. Additionally, the zoonotic potential of some of the pathogens isolated from IMI cases, the emergence of antibiotic resistance due to the extensive antibiotic use for IMI treatment, and the accumulation of antibiotic residues in milk and meat represent significant concerns for public health. Therefore, the investigation of IMI risk factors and the proposal of efficient measures to mitigate their effects on animal health and welfare is crucial. Although fly infestation is considered to play a significant role in the transmission of IMI pathogens, its adverse effects on udder health and the overall comfort status of dairy ewes have not been quantified and assessed on an evidential basis. Hence, the objectives of this study were to assess, for the first time, the fly repellent effect of deltamethrin and link it to: (i) the occurrence of common bacterial IMI; (ii) the somatic cell counts in milk; and (iii) the serum cortisol and creatine kinase levels (stress and fatigue indicators). The study was carried out in an intensive dairy sheep farm in northern Greece, during peak fly season. Deltamethrin treatment was associated with a reduced (i) number of flies (mostly *Musca domestica*) landing on treated ewes, compared to untreated ones (*p* < 0.05); (ii) colony-forming units in the case of Non-*aureus* Staphylococci IMIs (*p* < 0.05); and (iii) number of somatic cells in the milk (*p* < 0.001). Finally, serum cortisol and creatine kinase levels were significantly lower in deltamethrin-treated ewes (*p* < 0.001), indicating a less stressful environment for them.

## 1. Introduction

Intramammary infections (IMIs) consist a major issue in intensive dairy sheep production systems. They are mainly caused by bacterial infections [1], with their occurrence resulting in impaired udder health [2,3], decreased milk quantity and quality, and consequently, remarkable monetary losses at the flock level [4]. Moreover, IMIs have been identified as important causes of (i) welfare concerns in ewes [4]; and (ii) public skepticism regarding the safety and quality of milk and the products thereof [2]. The zoonotic potential of specific pathogens, antibacterial resistance due to the extensive use of antibiotics for the treatment of IMIs, and antibiotic residues in milk and meat, have emerged as public health hazards [5,6]. Nowadays, the existed legal and regulatory framework has not substantially described a comprehensive mitigation strategy against IMIs at the farm level in dairy ewes; instead, the extrapolation of data from dairy cattle is the norm, since studies to assess possible risk factors in dairy sheep are scarce.

Several microorganisms have been isolated from IMI cases in dairy ewes. Among them, *Staphylococcus aureus* is the most common cause of clinical mastitis [7], and non-*aureus* Staphylococci (NaS) are the most prevalent bacteria from subclinical mastitis (SCM) cases [7]. Other bacteria causing IMI include *Escherichia coli, Streptococcus* spp., *Mycoplasma* spp., *Trueperella pyogenes*, *Bacillus* spp., *Mannheimia haemolytica*, *Pseudomonas* spp., etc., whereas fungi could also contribute to the pathogenesis of IMIs [7]. In many cases, IMIs result in or predispose to mastitis [7], elevated somatic cell counts (SCC) in the milk [7,8] and decreased milk production [9].

The elucidation of possible risk factors, and modifications of health management and hygiene status facilitate the control of IMI at the flock level [10,11]. Factors such as the dysfunction of the milking machine, inappropriate milking practices, poor udder sanitary status, the absence of drying-off protocols and the inadequate hygiene of facilities and equipment have been proven to be of fundamental importance for the establishment of IMI [8,10].

Inadequate hygiene and inappropriate biosecurity measures, among others, predispose to fly infestation [12], which, in turn, further (i) deteriorate hygiene status, being directly linked to adverse rearing conditions inside the barns; and (ii) increases the possibility of intramammary infections, irritability, and stressful discomfort [12]. Moreover, flies usually disrupt normal feeding and stimulate defensive reflexes and behavior (i.e., head throwing, skin and tail twitching, reluctance to graze). Hence, they contribute to a stressful environment and decreased relaxation time (i.e., increased fatigue) due to the provoked annoyance they cause [13,14,15]. In addition to being annoying, horn flies (*Haematobia irritans*), sheep head flies (*Hydrotaea irritans*), stable flies (*Stomoxys calcitrans*) and common house flies (*Musca domestica*) can cause irritation and skin lesions, particularly in hairless parts, such as the udder and the genitals [12,14]. In addition, other fly species such as *Wohlfahrtia magnifica*, *Phormia regina* and *Lucilia cuprina* lay eggs or larvae on active wounds, leading to severe myiasis also known as “strike” [16].

Preventing health-threatening, stressful and fatigue conditions in farm animals is of paramount importance to enhance their health and welfare status and to satisfy consumer-driven demands for nutritious and safe foods derived from animals reared under welfare-friendly farming systems [17]. Several physiological, behavioral, clinical and biochemical traits have been proposed as direct or indirect indicators of impaired welfare status. Among them, serum cortisol (SC) and creatine kinase (CK) levels can be used as indicators of potential stressful and fatigue conditions. When animals are exposed to aversive or noxious stimuli (i.e., fly annoyance, painful bites), adrenal cortex response is activated, increasing the SC levels [17]. In addition, CK concentration in blood increases due to the leak of the enzyme in the blood stream, following muscle strain and damage after increased body exercise, disease or discomfort (restlessness due to heavy fly infestation) [18].

Therefore, the aim of the present study was to assess the association between deltamethrin treatment and (1) fly infestation; (2) the occurrence and severity of IMIs caused by common bacteria in sheep; (3) somatic cell counts in milk; and (4) SC as well as CK levels, in intensively reared dairy ewes, during peak fly season.

## 2. Results

### 2.1. Meteorological Data

During the study, climatic conditions at the location of the farm were typical (Thessaloniki, Central Macedonia, Greece) of the season (mid-June–mid-July 2020), according to the Hellenic National Meteorological Service; no significant variations in environmental temperature and relative humidity were observed between the three sampling occasions to affect the activity of flies. More precisely, at days 0, 15 and 30, the mean air temperature was 30.3 ± 2.3 °C, 32.4 ± 3.5 °C, and 31.2 ± 2.8 °C, respectively, and the mean relative humidity was about 41 ± 11.3%, 44 ± 17.6%, and 37 ± 13.1%, respectively.

### 2.2. Fly Species Identification

The most prevailing fly species, found on the sticky surface of the traps, were the common housefly, *Musca domestica* (89.9%), followed by the horn fly, *Haematobia irritans*, and the headfly, *Hydrotea irritans* (3.4 and 1.8%, respectively). Finally, other minor species were less commonly found (cumulative frequency 5.9%). The proportions of each fly genera, identified in FLY-REP (i.e., ewes individually dressed on their back with deltamethrin 10% after the morning milking, at day 0) and CON (i.e., ewes individually dressed on their back with a placebo treatment after the morning milking, at day 0) groups, did not differ significantly (*p* > 0.05), on days 0 and 30. At the same frame, the numbers of flies enumerated on the sticky traps did not differ between groups (*p* > 0.05), on the aforementioned sampling occasions.

### 2.3. Short and Long-Term Deltamethrin Repellency Effect

Table 1 summarizes the mean values (±standard deviation) and the comparisons between FLY-REP and CON groups as regards the number of flies landing on the ewes, for days 0 (both pre-treatment and 6 h post-treatment with deltamethrin), 15 and 30; in any case (except from day 0, pre-treatment), the number of flies landing on the ewes was significantly higher in the CON group compared to the FLY-REP group (*p* < 0.05). In addition, in the FLY-REP group, the population of flies was reduced within 6 h after the application of deltamethrin (on Day 0), whereas, in the CON group the number of flies remained unaffected.

### 2.4. The Association between Deltamethrin Treatment, Intramammary Infections, and the Somatic Cell Counts of Milk

In total, *S. aureus* was isolated from 58% (29/50), 64% (32/50) and 56% (28/50) of the assayed milk samples at days 0, 15, and 30, respectively. The respective numbers for NaS were 74% (37/50), 78% (39/50), and 86% (43/50), respectively. Overall mean values of *S. aureus* Colony Forming Units (CFU), NaS CFU and SCC were 5.2 ± 13.34 × 10^3^/mL, 4.6 ± 12.34 × 10^3^/mL and 2.1 ± 2.10 × 10^6^ cells/mL, respectively.

**Figure 1:**
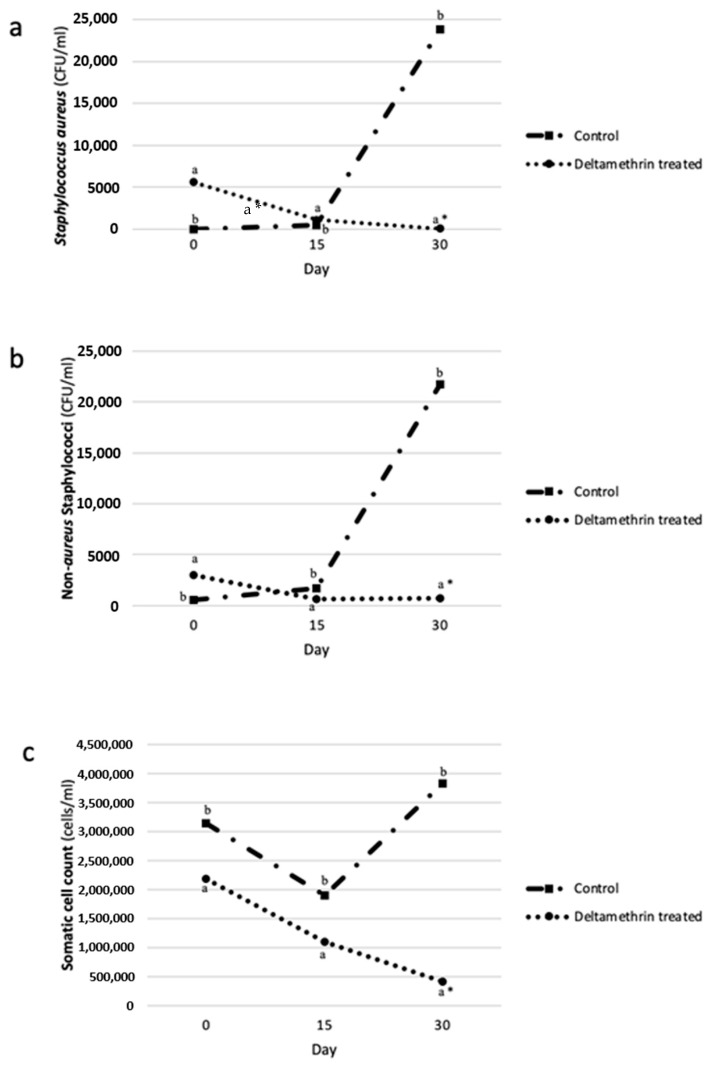
The evolution of (**a**) *Staphylococcus aureus* colony-forming units (CFU); (**b**) Non-*aureus* Staphylococci CFU; and (**c**) somatic cell counts, in the milk of deltamethrin treated (FLY-REP) and control group (CON), and comparisons between them (^a,b^ different superscripts in the same sample occasion indicate a statistically significant difference (*p* < 0.05) between the two groups; * statistically significant difference at the 0.001 level).

In the FLY-REP group, the prevalence of *S. aureus* IMIs for days 0, 15, and 30 was 72% (18/25), 68% (17/25), and 20% (5/25), whereas the prevalence of NaS IMIs for the same days was 56% (14/25), 72% (18/25), and 80% (20/25). In the CON group, the respective prevalence was 44% (11/25), 60% (15/25), and 92% (23/25) for *S. aureus* IMIs and 92% (23/25), 84% (21/25), and 92% (23/25) for NaS. The evolution of *S. aureus* CFU, NaS CFU and SCC, during the study, for the FLY-REP and the CON group, is presented in Figure 1. Additionally, Table 2 summarizes the areas under the curve (AUC) of the two groups, and the comparisons between them, for *S. aureus* and NaS CFU and for SCC in milk. AUC was significantly higher in the CON group for (i) *S. aureus* CFU (*p* < 0.001); (ii) NaS CFU (*p* < 0.001); and (iii) SCC (*p* < 0.05) between 15 and 30 days and 0 and 30 days.

Based on the mixed linear model results, and after accounting for the random effect of the ewe and the fixed effect of sampling occasion, it was evidenced that deltamethrin treatment was associated with reduced LogNaS CFU and LogSCC (Table 3). Specifically, in the FLY-REP group LogNaS CFU and LogSCC were reduced by 0.44 (*p* < 0.05, 95% CI, 0.10 to 0.79) and 0.53 (*p* < 0.01, 95% CI, 0.26 to 0.8) logarithms compared to the CON group. Deltamethrin treatment was not significantly associated with Log*S.aureus* CFU (Table 3).

The association between deltamethrin treatment and *E. coli* and *Mycoplasma* spp. IMIs was not possible to be estimated in our study, due to the low prevalence of *E. coli* (1.3%, 2/150). *Mycoplasma* spp. strains were not isolated in any case.

### 2.5. The Association between Deltamethrin Treatment, Serum Cortisol (SC) and Creatine Kinase (CK) Levels

**Figure 2:**
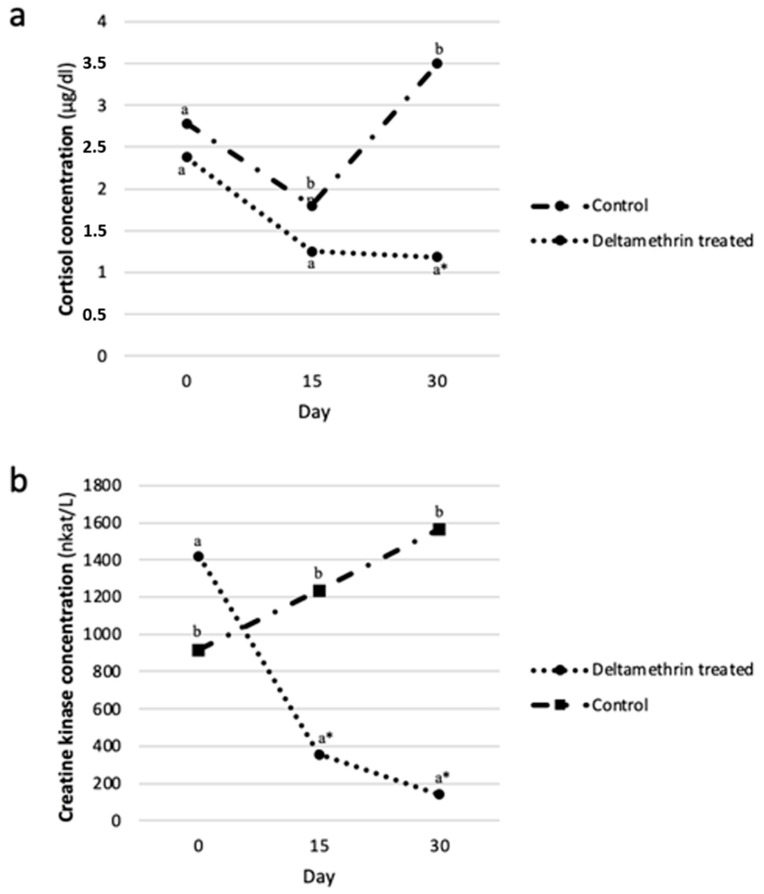
The evolution of serum (**a**) cortisol (μg/dL); and (**b**) creatine kinase (nkat/L) in the deltamethrin-treated (FLY-REP) and control group (CON), and comparisons between them (^a,b^ different superscripts in the same sampling occasion indicate statistically significant difference (*p* < 0.05) between the two groups; * statistically significant difference at the 0.001 level).

Overall, the mean values of SC and CK in the ewes included in the study were 2.2 ± 0.98 μg/dL, and 954.8 ± 800.61 nkat/L (or 57.3 ± 48.04 IU/L). Figure 2 illustrates the evolution of SC and CK levels, during the study, for the ewes in the FLY-REP and CON groups. The mean (± standard deviation) values of the AUC for the two groups, and comparisons between them, for SC and CK levels, are presented in Table 4.

According to the mixed linear and the inverse Gaussian regression model used to estimate the effect of deltamethrin treatment on SC and CK, respectively, it was found that deltamethrin treatment was associated with a significant decrease in the SC and CK levels (Table 3); namely, in the FLY-REP group, the logarithms of SC and CK levels were reduced by 0.44 (*p* < 0.05, 95% CI, 0.10 to 0.79) and 0.53 (*p* < 0.01, 95% CI, 0.26 to 0.8) compared to the CON group.

## 3. Discussion

The aim of our study was to assess the fly repellent effect of deltamethrin treatment, as well as its association with (i) the IMI caused by *S. aureus* and NaS; (ii) the SCC of milk; and (iii) the serum cortisol and creatine kinase levels in intensively reared dairy ewes, during peak fly season.

The warm and temperate climate of Greece favors fly infestation, particularly during the hot–humid season of the year (i.e., spring and early summer) [14]. During this period, flies utilize the optimal environmental conditions within the livestock enterprises (e.g., moisture, manure accumulation, inappropriate hygiene, etc.) to reproduce quickly. In our study, the mean temperature (30 to 32 °C) and relative humidity (37 to 44%) in the region of the studied farm were characteristic of the season (i.e., June and July) and favored the survival and rapid increase in the fly population. This is the norm in dairy sheep farms across Greece, with fly infestation being considered a major issue disrupting comfort conditions for the animals and humans and undermining hygiene status at the farm level.

To our knowledge, the identification of the most prevalent fly species in dairy sheep farms was attempted for the first time. As expected, the common housefly was the predominant fly species detected (ca. 90%). These flies are not only a source of annoyance, but they are also carriers of viruses, bacteria, helminths, and protozoa, therefore, playing a crucial role in their transmission [19,20,21,22]. The horn and the head fly were the second and the third more frequently observed fly species. Although their frequency was very low in comparison to the common housefly (3.4 and 1.8%, respectively), this is a remarkable finding as these flies: (i) feed on blood, sweat, saliva, skin secretions of the animals they infest, either by puncturing the skin or by scavenging on the skin surface or wounds; (ii) inflict painful bites while blood-feeding; and additionally (iii) serve as mechanical and biological vectors for a wide variety of pathogens [14]. The scarcity of relevant studies in the available literature renders impossible the extensive benchmark of the observed fly population regarding its density and composition; two factors which are significantly affected by the global variety of fly species, climatic conditions, farming systems and the available biosecurity measures. For example, according to Sajid et al. [23] the biting fly, *Stomoxys calcitrans*, is the only species found infesting domestic sheep in Pakistan.

Reduction in the fly population at the farm level is a challenging endeavor. For this reason, practices such as sanitation and fly exclusion, insecticides, and fly traps are utilized inside and around facilities [24]. Additionally, fly repellents are currently exploited, with deltamethrin being one of them. Deltamethrin is a highly effective insecticide, belonging to the group of synthetic pyrethroids. Despite the fact that it is used for the control of several ectoparasites, including different species of flies [25], the scientific evidence of its efficiency against fly infestation in dairy sheep has not been sufficiently documented. In our study, this was the first time that the application and effectiveness of deltamethrin as a fly repellent in dairy ewes was studied, and the beneficial effects against fly infestation evidenced.

Among the pathogens that can be transferred by flies, coagulase positive Staphylococci are of major interest, being the most commonly involved in IMIs and the pathogenesis of mastitis. Field reports have indicated that these staphylococci have been isolated from up to 70% of cases of clinical mastitis in dairy sheep flocks [26,27,28,29]. According to Bergonier et al. [7], *S. aureus* is the most frequently diagnosed etiological agent of clinical mastitis in dairy ewes. It is normally found on the skin of animals and other surfaces causing IMI [30] which can conditionally lead to mastitis; the severity of *S. aureus*-derived mastitis depends on the initial bacterial load, the virulence of the strain, and the immune response of the infected animal [31]. According to Bergonier and Berthelot [26], the incidence is probably less than 7% across a lactation, while Al-Majali and Jawabrech [28] reported that sporadically, *S. aureus* may account for as many as 40% of isolates from cases of clinical mastitis. In our study, *S. aureus* isolation ranged from 56 to 64% of the samples collected but was not associated with the occurrence of clinical mastitis. This is not surprising, as *S. aureus* strains are commonly isolated from cases of subclinical mastitis or from cases of IMIs which are not followed by the inflammation of the mammary gland [29].

Vectors, like flies, transmit several pathogens, including *S. aureus*, among farm animals. Although the role of flies in pathogen transmission has been described in dairy cattle [19,20,21,22], until now there has been no systematic appraisal of their role in dairy sheep flocks; therefore, the extrapolation of data from dairy cattle was attempted to better understand and handle the problem. Although in cows flies are considered a potential vector involved in the horizontal transmission of *S. aureus* [19,20,22], in our study, an association of deltamethrin treatment with *S. aureus* isolation rate from IMI cases was not evidenced.

In opposition, for the first time, we found that deltamethrin application was associated with decreased CFU of NaS, isolated from IMI cases. Despite NaS being considered as minor, opportunistic pathogenic bacteria, their significance has been proven to be noticeable for the udder hygiene [7], as they can constitute up to 70% of the bacterial isolates from cases of subclinical mastitis [2]. In our study, NaS have been isolated from the collected milk samples at rates ranging from 74 to 86%. prevent possible contamination and misleading results, sample collection was performed by an experienced veterinarian, according to standard sampling protocols to prevent the contamination of the collected samples, as described in the Materials and Methods. Although NaS were not identified at the species level, the estimation of the association of deltamethrin treatment with the isolation rate of IMI-causing NaS is of great value for the cost–benefit analysis when designing biosecurity and udder health management protocols. This finding is in accordance with several studies in cattle which have proven the major role of flies on the transmission of NaS among hosts, especially during summer [32,33,34].

In the present study, only two strains of coliforms (*E. coli*) were isolated. The observed frequency is closer to the lower limits (about 1.3%) of the available literature, where coliforms account for 1.4 to 14.2% of the IMI in the sheep flocks [27]. This is a rather expected finding as *E. coli*, and coliforms in general, are commonly isolated from IMI in dairy cattle but not in dairy ewes [35,36], due to the drier consistency of sheep feces comparing to cattle [8]. The lower prevalence of *E. coli* IMI did not allow the assessment of the association between deltamethrin treatment and *E. coli* CFU; therefore, further studies, potentially involving farms with increased prevalence of *E. coli* IMI, are needed.

Other factors, affecting the excretion rate of microorganisms (i.e., bacteria) with the milk of the infected mammary glands, are associated with immunomodulatory situations provoked by stress. Periparturient stress is the most critical stage of the production cycle of dairy ewes as the combination of parturition, increasing milk yield and low dry matter intake compromise host defenses, allowing the excretion of high numbers of microorganisms. Nutritional stress should be considered as a factor allowing increased microbe excretion. When the nutrient supply (i.e., energy, protein, macro-micro elements and vitamins deficiency) is not adequate for the body maintenance and milk production, dairy ewes prioritize the providing nutrient supply, overpassing the needs of the immune system. Climatic/environmental stress has also been documented to affect the mammary carriage. Sudden changes in the temperature–moisture level or moving away from the animals’ accustomed settings increase the colony forming units in milk samples [37,38]. Milking practices, general hygiene and biosecurity measures, and the overall herd health management protocol (e.g., intramammary antibiotic treatment at dry period, vaccination against *S. aureus*) seem to interact with mammary carriage. Finally, the breed of dairy ewes, the number and stage of lactation have also been found to affect IMI [2,3,29].

Subclinical mastitis is defined as the intramammary infection in the absence of clinically detectable changes. The best method for the detection of the subclinical inflammatory reaction in the mammary gland remains the demonstration of increased SCC in the milk [36]. However, in contrast to the cows’ milk, in sheep milk, the thresholds of SCC, beyond which mammary inflammation is suspected, have not yet been established in a universally accepted manner [39]. According to Berthelot et al. [40], the enumeration of less than 500,000 cells/mL of milk indicate a healthy mammary gland (i.e., the absence of clinical or subclinical mastitis), which does not require laboratory confirmation. On the contrary, milk with more than 1,000,000 cells/mL is considered to originate from a mammary gland with either subclinical or clinical mastitis and confirmative bacteriological examination is necessary [40]. In our study, we estimated that SCC and bacteriological exams were performed to investigate IMI cases; however, the profile of cells (epithelial, macrophages, neutrophils, lymphocytes, etc.) was not determined, therefore, the definitive diagnosis of mastitis, either in its clinical or subclinical form, was not possible [36,41]. In any case, the increased overall mean value of SCC (ca. 2 × 10^6^ cells/mL) is indicative of the increased prevalence of IMI and mastitis [40]. This was an expected finding, based on the history of the flock, which despite the control measures adopted in recent years, the problem still existed. The reduction in the logarithm of SCC by 0.53 in the deltamethrin-treated group is an interesting finding, leading to the assumption that deltamethrin treatment is associated with improved mammary gland health status via the reduction in pathogens transferred by flies and the inflammation caused by them.

Except from udder health status, fly infestation is a major challenge for dairy sheep welfare [42]. The licking and blood sucking activity of the flies cause discomfort, annoyance, pain, and significantly affect the behavior of the ewes, promoting behavioral responses which configure a stressful situation for the animals. Several studies which investigated behavioral alterations in dairy and beef cattle infested by flies, have demonstrated the lineal correlation between heavy fly infestation and increased defensive behaviors [43,44,45]; relevant studies in dairy ewes are scarce.

Currently, there is increasing attention regarding the quantification of stress levels, which is directly linked with welfare and health status [46,47]. Serum cortisol has been promoted as a widely used indicator for the assessment of stress in cattle [48]. In this species, among the studied factors which affect the SC level, flies have been found to play a significant role. Indeed, Schwinghammer et al. [49] reported a high serum cortisol level due to the heavy infestation of beef cattle by stable flies. Similarly, Vitela-Mendoza et al. [45] concluded that the plasma cortisol level is linearly related to the number of flies in dairy cattle herds. This relationship has never been described in dairy ewes. This is the first study investigating the association between deltamethrin treatment and the SC; lower levels of SC were observed in the treated ewes. It could be assumed that the use of deltamethrin facilitates the better control of flies and the formation of a more welfare-friendly environment for the ewes. To prevent the overestimation of stress levels, factors such as the circadian rhythm of the studied animals, need to be considered when measuring their stress status [48].

In addition to the SC, deltamethrin treatment was associated with a lower CK level. Fly infestation makes animals hypersensitive and increases the muscular exertion and damage [50,51,52] due to decreased resting time and increased defensive behaviors and activity. Under these circumstances, blood CK levels increase, indicating the fatigue status of the animals [18]. To date, studies supporting the link between fly burden in farm animals and fatigue status as determined by the CK level have not been published. Therefore, this study can be used as a paradigm towards similar studies in which other treatments, practices and conditions could be assessed in terms of their contribution to a more restful environment.

A study design where the pen would be used as the experimental unit would be of interest, as it would enable addressing the confounding effect of the pen within the treatment. In the current study, a limited confounding effect of the pen was expected, as the variation explained by the pen itself was not expected to be remarkable (the two pens were appropriately designed to be similar and were allocated in the same area; also breed, feeding, age, lactation stage, milk yield and the overall management of the animals were similar in the two pens).

## 4. Materials and Methods

### 4.1. Flock History

For the study, a commercial, intensive dairy sheep flock consisting of 800 dairy ewes of Lacaune breed, and located at Thessaloniki (Central Macedonia, Greece) was used. The flock had a history of increased incidence of subclinical/clinical mastitis, especially during spring and early summer, when fly population was dense. Housing facilities of the farm allowed an adequate resting area (2.2 m^2^/ewe), as well as appropriate feeding (0.41 m/ewe) and drinking space per ewe. During the study, the animals were fed a typical diet including 1.0 kg of concentrates (divided into three meals and fed in the milking parlor) and the same quantity of alfalfa hay on a daily basis; access to wheat straw and water was ad libitum. The average annual milk production per ewe was approximately 380 L. Ewes were vaccinated for clostridial diseases, approximately 20 days before parturition and 6 months later. Vaccination against *Mycoplasma agalactiae* was performed 30 days before parturition and after 6 months. The endoparasite control strategy included the *per os* administration of benzimidazole (10 mg/kg of body weight), once per year, at lambing. None of the ewes participating in the study were treated using antibiotics in the last 30 days before the initiation of the study.

### 4.2. Experimental Design

This was a prospective study conducted between June and July 2020. A total of fifty multiparous ewes, aged between 3 and 4 years, at mid-lactation period (i.e., 4–6 months post-partum), were randomly selected and included in the study. The animals were selected from the same group, therefore, except from being at the same stage of lactation, they were also fed the same diet, and had a similar daily milk yield. After the initial selection, the animals were randomly assigned into two groups of 25 ewes each, coded as the FLY-REP and CON groups. In the FLY-REP group, the ewes were individually dressed on their back with a solution of deltamethrin 10% (Deltanil^®^ 10 mg/mL, Virbac Hellas, Greece) after the morning milking, at day 0. Deltamethrin was applied only once, in accordance with the manufacturers’ instructions Figures S1–S3. The same day, a placebo treatment was also applied at the back of the ewes in CON group. The treatment of ewes started with the CON group and was followed by the FLY-REP. For the study, each group was kept in a separate pen to prevent any accidental transfer of the drug.

### 4.3. Milking Routine and Technical Information of the Milking Machine

Due to the mastitis history of the farm, a detailed milking protocol was developed in the farm, and important milking practices were routinely followed. These included: (i) the wearing of disposable latex gloves; (ii) pre-stripping to stimulate the milk-ejection reflex and check for milk abnormalities; (iii) detachment of the clusters following vacuum release; (iv) post-stripping on an individualized basis; (v) post-dipping the teats with 1.5% iodine solution (Desintec^®^ MH-Iodine, Agravis, Germany), to disinfect the teats and prevent the inoculation of bacteria into the teat canal; and (vi) the use of alkaline and acid detergents (Desintec^®^ MH-Iodine, Agravis, Germany), for the cleaning and disinfection of the milking machine after every milking, according to the manufacturer’s instructions.

A 2 × 24 parallel milking parlor (Westfalia Technologies, Larisa, Greece) was used and set at 38 kilopascals, 180 cycles per minute, and 60/40 regarding vacuum level, pulsation rate and pulsation ratio, respectively. The functionality and performance of the milking parlor was assessed every four months by specialized technical staff, during routine service. The liners had been replaced one month before the initiation of our study.

### 4.4. Fly Monitoring, Trapping and Identification

The number of flies, landing on the ewes of both groups, was counted by the direct observation of each individual animal. The accuracy of this procedure and inter-observer reliability have been well documented by relevant studies [53,54,55]. Fly populations were counted on half of the ewes’ body from the neck to the tail and from the spinous processes of the vertebrae to the belly and the feet [53]. The same observer recorded the number of flies for each individual animal from a distance of approximately 3 meters, for 2 min, on days 0, 15 and 30 between 11:00 p.m. and 02:00 a.m. On day 0, an additional counting was done 6 h post-treatment, for the assessment of the short-term deltamethrin repellency effect. The observation of animals and fly monitoring was in compliance with animal welfare rules and did not cause any significant stress to the animals.

For the study of fly population, ten fly traps with a sticky surface (Fly Catcher trap, Zhejiang, China, roll of 5 × 120 cm) were placed at equal distances in the pen of each one of the two groups on days 0 and 30. The traps remained in the pens for 24 h and after their use, they were put into separate plastic containers and transferred to the Laboratory of Parasitology and Parasitic Diseases of the School of Veterinary Medicine of the Aristotle University of Thessaloniki, Greece, where they were stored in closed containers in a 70% ethanol and 30% glycerol solution. Fly species were identified according to morphological keys proposed by Wall and Shearer [56] and Couri et al. [57] and their frequency was estimated by counting.

### 4.5. Meteorological Data

Meteorological data for Thessaloniki (Central Macedonia, Greece) were acquired by the Hellenic National Meteorological Service. Mean air temperature and relative humidity of the farm region per sampling occasion (days 0, 15 and 30) and during the observation were calculated for the assessment of possible effects of environmental conditions, on fly population and/or activity.

### 4.6. Milk Sampling and Analyses

On every sampling occasion, milk samples were collected from each individual ewe following standard milk sampling protocol. In brief, teats were properly disinfected using 90% isopropyl alcohol-soaked wipes, the first 3 squirts of milk were discarded, and a milk sample was collected in a sterile tube, by milking approximately equal volumes of milk from each udder half. After the sample collection, teats were disinfected with a solution of 1.5% iodine (Desintec^®^ MH-Iodine, Agravis, Germany).

Afterwards, the milk samples were placed into isothermal container at approximately 4 °C and were transported to the “Vet-Analyseis” Veterinary Microbiological Laboratory in Larissa (Thessaly, Greece) to be microbiologically assayed as described below.

As soon as the milk samples arrived in the lab, 50 μL of undiluted milk from each one of them was suffused on sheep blood agar (Oxoid Company, Hampshire, UK) and incubated under aerobic conditions (i.e., 37 ± 1 °C for 22 ± 2 h). A milk sample was considered contaminated when ≥3 different bacterial species were isolated. Additionally, 50 μL of undiluted milk from each milk sample was plated on mannitol salt agar (Oxoid Company, Hampshire, UK) and McConkey agar (Biolife Company, Milan, Italy). The process was repeated after one ten-fold dilution of each milk sample. At the end of the incubation period (i.e., 37 ± 1 °C for 48 ± 2 h), bacterial cultures were identified according to the morphology of the colony, Gram-staining, catalase reaction and other biochemical reactions such as the activity of DNAse, coagulase test, pattern of hemolysis, reaction of lactose, catalase and indole and finally, the hydrolysis of esculin. In mannitol salt agar, the cultivated Gram-positive cocci were differentiated with catalase reaction into catalase-positive and catalase-negative cocci. *S. aureus* isolates were differentiated from NaS, based on DNAse activity, colony morphology, coagulase tube test result and the pattern of hemolysis. In McConkey agar, oxidase negative *E. coli* were discriminated from other coliforms by their positive reaction to the lactose, catalase and indole test, performed on 44 °C. Finally, streptococci, which reacted negatively to catalase and esculin test in sheep blood agar, were identified based on their hemolytic properties. Colonies (CFU/mL), which were cultivated on mannitol salt and McConkey agars, were enumerated for the estimation of the staphylococci (i.e., *S. aureus* and NaS) and *E. coli* populations [58].

The differentiation of other less important milk contaminants including *Enterobacter* spp., *Citrobacter* spp., *Mannheimia* spp., *Proteus* spp. and fungi, at the genus level, was performed with conventional methods [59]. All Enterobacteriaceae colonies which were reacted negative or positive/negative to the oxidase and lactose test, respectively, were further cultured from McConkey agar to the Nutrient agar (Oxoid Company, Hampshire, UK). At the same frame, Gram-negative, oxidase-positive bacteria were subcultured from the sheep blood agar to the Nutrient agar. After the end of incubation time, the aforementioned bacteria were identified with the help of two different identification systems, GN-A and GN-B (Microgen Company, Surrey, UK). The first one is a system of identification for the most widely isolated Enterobacteriaceae, containing twelve substrates, while the second one contains twenty-four substrates.

Furthermore, the presence of *Mycoplasma* spp., in each milk sample, was evaluated with the use of Eaton’s agar and broth, which were prepared in the laboratory. In brief, 0.1 mL of each undiluted milk sample was streaked on Eaton’s agar [60] and incubated for 3–7 days at 37 ± 1 °C in a humidified incubator supplied with 5% CO_2_. Meanwhile, 0.5 mL of undiluted milk, taken from each milk sample, was incubated in 10mL Eaton’s broth [56] at 37 ± 1 °C under aerobic conditions. Broth-to-broth passages and subcultures to agar plates were made twice at 48 to 72 h intervals. Given the fact that no isolates of *Mycoplasma* spp. were found, further molecular assays were not applicable.

Finally, somatic cell counts (SCCs) were estimated in each milk sample using an automatic high-throughput analyzer (FossomaticTM FC, Foss Analytical A/S, Hillerød, Denmark).

### 4.7. Blood Sampling and Analyses

Blood samples were collected by jugular venipuncture, in a BD Vacutainer^®^ tube without anticoagulant, from each individual ewe, on days 0, 15 and 30. For blood collection, the ewes were humanely restrained in the milking parlor and the blood sample was taken by an experienced vet. Blood samples were transported to the lab within 2 h after sampling and the serum was separated, through centrifugation at 1000 rpm for 15 min, at environmental temperature. Serum samples were frozen (−20 °C) until the SC and CK concentration was measured. The estimation of cortisol concentration was performed with electrochemiluminescence immunoassay method (ECLIA), using Roche Cobas E601 (Diamond Diagnostics, Holliston Massachusetts, USA) immunology analyzer. At the same frame, the estimation of CK concentration was performed with spectrophotometric method, using the Roche Cobas C501 (Diamond Diagnostics, Holliston Massachusetts, USA) chemistry analyzer. The precision of the forementioned methods as characterized by the inter- and intra-coefficient of variation (CV%) for the determination of SC level in serum samples were 3.8 and 2.6%, respectively, while values for CK level were 3.4 and 2.2%, respectively.

### 4.8. Statistical Analysis

All statistical analyses were performed using SPSS v23 (IBM, New York, NY, USA) and included both descriptive (mean ± standard deviation and frequencies for continuous and categorical variables, respectively) and analytical statistics. Shapiro–Wilk and Levene’s test were used to check the assumptions of normality and homogeneity of variances, respectively, for the continuous variables. In cases, where the assumption of normality was not satisfied, the log transformation of the data was applied; thereby, *S. aureus* CFU, NaS CFU, SCC, as well as SC and CK concentrations were log transformed (Log*S.aureus* CFU, LogNaS CFU, LogSCC, LogSC, and LogCK, respectively.

Initially, Wilcoxon rank sum test (Mann–Whitney U test) was used to compare the FLY-REP and CON groups regarding (i) the number of flies landing on the ewes; (ii) the number of *S. aureus*, NaS (CFU/mL) and SCC (cells/mL); and (iii) SC and CK levels on days 0, 15 and 30. Chi-square test used to compare the frequencies of each of the fly genera at days 0 and 30.

Areas under the curves (AUC) were calculated and one-way ANOVA with Bonferroni’s correction was used to compare AUC of the two groups for (i) *S. aureus*, and NaS CFU; (ii) the SCC from the collected milk samples; and (iii) SC and CK levels between days 0 to 15, 15 to 30, and 0 to 30.

Afterwards, mixed linear regression models were built to estimate the random effect of the k^th^ ewe and the fixed effects of deltamethrin treatment and s^th^ sampling occasion on the logarithmic values of *S. aureus* CFU, NaS CFU, SCC, as well as SC and CK levels, as described below:

Y_sk_ = μ + D_k_ + G_k_ + γ_k_ + e_k_ (Model 1) (1)

where: Y_sk_= dependent variable (Log*S.aureus* CFU, LogNaS CFU, LogSCC, LogSC, and LogCK), μ= intercept, T_k_ = fixed effect of deltamethrin treatment (2 levels, 0 = treated with deltamethrin, 1 = control), G_k_ = fixed effect of the sampling occasion (3 levels, days 0, 15 and 30), γ_k_ = repeated variation of the k^th^ ewe, and e_k_ = residual error.

Due to a significant positive skewness in the logarithmic values and the residuals of the model estimating the Log CK, an inverse Gaussian regression model with the same predictors was used. In every case, the first order autoregressive was used as covariance structure. Statistical significance was set at the 0.05 level.

## 5. Conclusions

The present study, conducted in one dairy sheep flock of Greece, recorded for the first time that deltamethrin application was associated with decreased colony forming units of NaS, isolated from IMI cases. This is a significant finding which should be taken under consideration when designing biosecurity and udder health management protocols. It is worth noting that the reduction in the logarithm of SCC in the deltamethrin-treated group is another novel finding, leading to the assumption that fly repellency using deltamethrin may have an indirect effect on the health status of the mammary gland health via the reduction in pathogens transferred by flies and the inflammation caused by them. Furthermore, this is the first study investigating the association between deltamethrin treatment and the SC and CK concentrations, which were found lower in the treated ewes. It could be assumed that the use of deltamethrin facilitates the better control of flies and contributes to the formation of a more welfare-friendly environment for intensively reared dairy ewes.

## Figures and Tables

**Table 1:** Mean number (± standard deviation) of flies, landing on dairy ewes, in the deltamethrin-treated group (FLY-REP) and the control group (CON) on days 0 (pre-treatment and 6 h post-treatment), 15 and 30, and comparisons between the two groups.

Day		Group	
		FLY-REP	CON
0	(Pre-Treatment)	45.0 ^a^ (± 11.04)	53.0 ^a^ (± 6.01)
	(6 Hours Post-Treatment)	7.4 ^a^ (± 4.12)	78.0 ^b^ (±1 9.83)
15		4.4 ^a^ (± 1.34)	66.5 ^b^ (± 28.22)
30		7.3 ^a^ (± 2.52)	69.8 ^b^ (± 12.34)

^a,b^ Different superscripts in the same row indicate statistically significant differences between the two groups (*p* < 0.05).

**Table 2:** Mean (±standard deviation) areas under the curve (AUC) of the deltamethrin-treated group (FLY-REP) and the control group (CON), and comparisons between them, for (i) *Staphylococcus aureus* and Non-*aureus* Staphylococci intramammary infections (CFU × 10^3^/mL); and (ii) somatic cell counts in milk (somatic cells × 10^6^/mL), throughout the study.

Days	AUC _FLY-REP_	AUC _CON_
*Staphylococcus aureus* (CFU × 10^3^/mL)		
0–15	50.2 ^a^ (± 4.99)	3.7 ^b^ (± 0.69)
15–30	8.8 ^a^* (± 3.50)	182.2 ^b^ (± 34.67)
Total	59.9 ^a^* (± 2.93)	185.9 ^b^ (± 126.22)
Non-*aureus* Staphylococci (CFU × 10^3^/mL)		
0–15	27.2 ^a^ (± 11.44)	17.2 ^a^ (± 5.44)
15–30	10.0 ^a^* (± 1.20)	175.9 ^b^ (± 12.46)
Total	37.2 ^a^* (± 12.17)	193.1 ^b^ (± 112.19)
Somatic Cells Count (Cells×10^6^/mL)		
0–15	24.7 ^a^ (± 16.60)	37.9 ^b^ (± 12.22)
15–30	11.4 ^a^ (± 0.59)	43.0 ^b^ (± 21.06)
Total	36.1 ^a^ (± 9.39)	80.8 ^b^ (± 23.60)

^a,b^ Different superscripts in the same row indicate statistically significant differences between the two groups (*p* < 0.05); * statistical significant difference at the ≤ 0.001 level.

**Table 3:** Associations between deltamethrin treatment and the sampling occasion as estimated by the mixed linear regression models on the logarithms of milk somatic cell counts, and colony-forming units of *Staphylococcus aureus* and Non-*aureus* Staphylococci; serum cortisol (SC) and creatine kinase (CK) levels, in the studied ewes.

Parameter	Category Level	*B*	SE	*p*-Value	95% CI	
					Lower	Upper
Logarithm of *Staphylococcus aureus* Colony-Forming Units (Mixed Linear Regression)						
Deltamethrin Treatment	Yes	0.13	0.176	0.459	−0.23	0.49
	No	*“Ref”*				
Sampling Occasion	Day 0	−0.32	0.210	0.135	−0.74	0.10
	Day 15	−0.88	0.198	0.000	−1.28	−0.49
	Day 30	*“Ref”*				
Intercept	Continuous	3.68	0.144	0.000	3.39	3.97
Logarithm of Non*-aureus Staphylococci* Colony-Forming Units (Mixed Linear Regression)						
Deltamethrin Treatment	Yes	−0.44	0.171	0.014	−0.79	−0.10
	No	*“Ref”*				
Sampling Occasion	Day 0	−0.60	0.151	0.000	−0.90	−0.30
	Day 15	−0.58	0.127	0.000	−0.83	−0.32
	Day 30	*“Ref”*				
Intercept	Continuous	3.59	0.139	0.000	3.31	3.87
Logarithm of Milk Somatic Cell Counts (Mixed Linear Regression)						
Deltamethrin Treatment	Yes	−0.53	0.107	0.003	−0.80	−0.26
	No	*“Ref”*				
Sampling Occasion	Day 0	0.36	0.080	0.000	0.20	0.51
	Day 15	0.09	0.069	0.200	−0.05	0.23
	Day 30	*“Ref”*				
Intercept	Continuous	6.16	0.150	0.012	4.55	7.78
Serum Cortisol Level (Mixed Linear Regression)						
Deltamethrin Treatment	Yes	−0.30	0.033	0.000	−0.36	−0.23
	No	*“Ref”*				
Sampling Occasion	Day 0	0.12	0.030	0.000	0.07	0.18
	Day 15	−0.08	0.026	0.003	−0.13	−0.03
	Day 30	*“Ref”*				
Intercept	Continuous	0.43	0.028	0.000	0.37	0.48
Creatine Kinase Level (Inverse Gaussian Linear Regression)						
Deltamethrin Treatment	Yes	−0.58	0.037	0.000	−0.65	−0.51
	No	*“Ref”*				
Sampling Occasion	Day 0	0.53	0.079	0.000	0.37	0.68
	Day 15	0.16	0.054	0.003	0.05	0.26
	Day 30	*“Ref”*				
Intercept	Continuous	2.88	0.046	0.000	2.79	2.97

CI: confidence interval (Wald confidence interval was calculated for the inverse Gaussian model); *B*: coefficient; SE: standard error; *“Ref”*: reference category. In the control group, the mean estimated values (±standard error) of the logarithms of *Staphylococcus aureus* and Non-*aureus* Staphylococci colony-forming units and milk somatic cell counts were 3.3 ± 0.18, 3.2 ± 0.17, and 6.3 ± 0.11, respectively; the mean estimated values of the logarithms of SC and CK levels were 0.44 ± 0.03 and 3.11 ± 0.04, respectively.

**Table 4:** Mean (±standard deviation) areas under the curve (AUC) of the deltamethrin-treated group (FLY-REP) and the control group (CON), and the comparisons between them, for serum cortisol, and creatine kinase level, throughout the study.

Days	AUC _FLY-REP_	AUC _CON_
Cortisol (μg/dL)		
0–15	27.2^a^ (± 4.55)	34.4^a^ (± 18.05)
15–30	18.2^a^ (± 2.34)	32.8^b^ (± 11.57)
Total	45.4^a^ (± 6.36)	67.1^b^ (± 18.13)
Creatine Kinase (× 10^3^ nkat/L)		
0–15	13.3^a^ (± 2.30)	16.1^a^ (± 0.45)
15–30	3.7^a^ (± 0.67)	21.0^b^ (± 8.67)
Total	17.1^a^ (± 6.79)	37.1^b^ (± 3.44)

^a,b^ Different superscripts in the same row indicate statistically significant differences between the two groups (*p* < 0.05).

## Supplementary Materials

The following are available online at https://www.mdpi.com/2076-0817/10/2/232/s1, Figure S1: Separation of the wool of the ewe before the application of deltamethrin (Deltanil^®^, Virbac), Figure S2: Application of deltamethrin (Deltanil^®^, Virbac) and Figure S3: The area of deltamethrin (Deltanil^®^, Virbac) application post-treatment.
