# Supplementary material for: Will Fly Repellency Using Deltamethrin Reduce Intramammary Infections, Stress and Fatigue Indicators of Dairy Ewes under Intensive Management?
Source: Pathogens. 2021 Feb 19;10(2):232. doi: 10.3390/pathogens10020232 (PMC7922805; doi:10.3390/pathogens10020232)
Supplement: Supplementary file 1 [file pathogens-10-00232-s001.pdf]

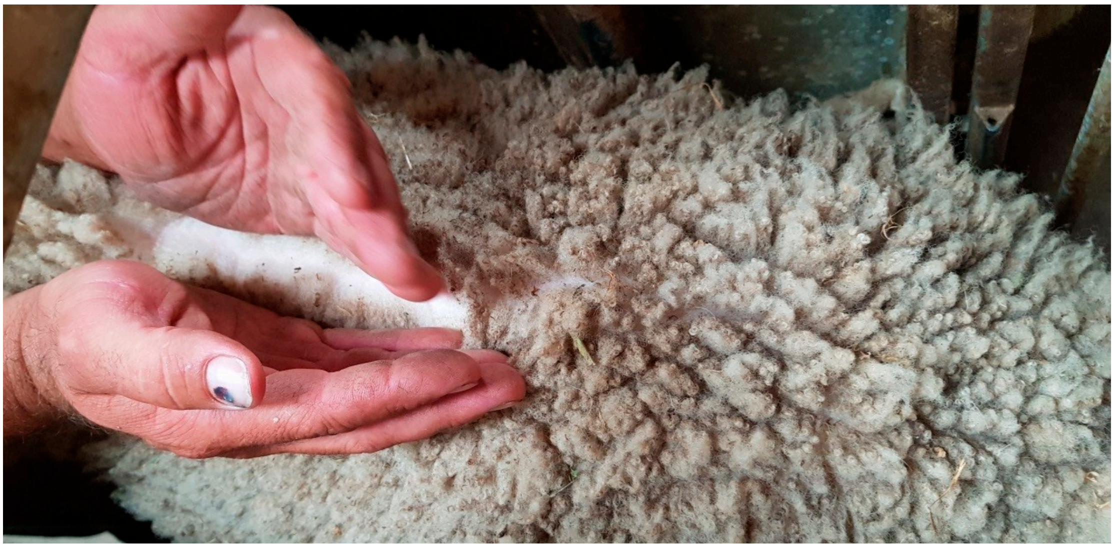

Figure S1. Separation of the wool of the ewe before the application of deltamethrin (Deltanil®, Virbac).

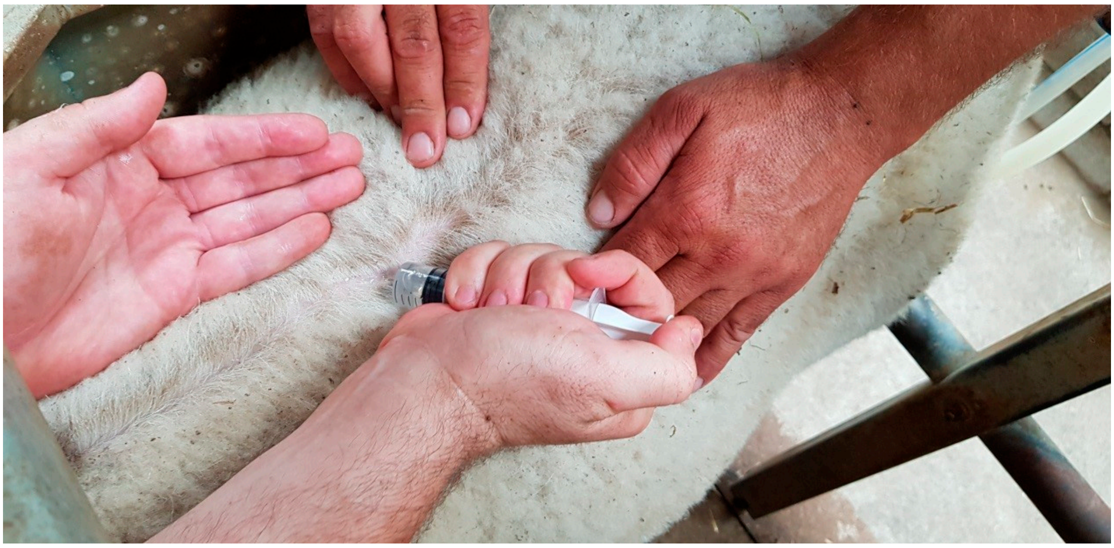

Figure S2. Application of deltamethrin (Deltanil®, Virbac).

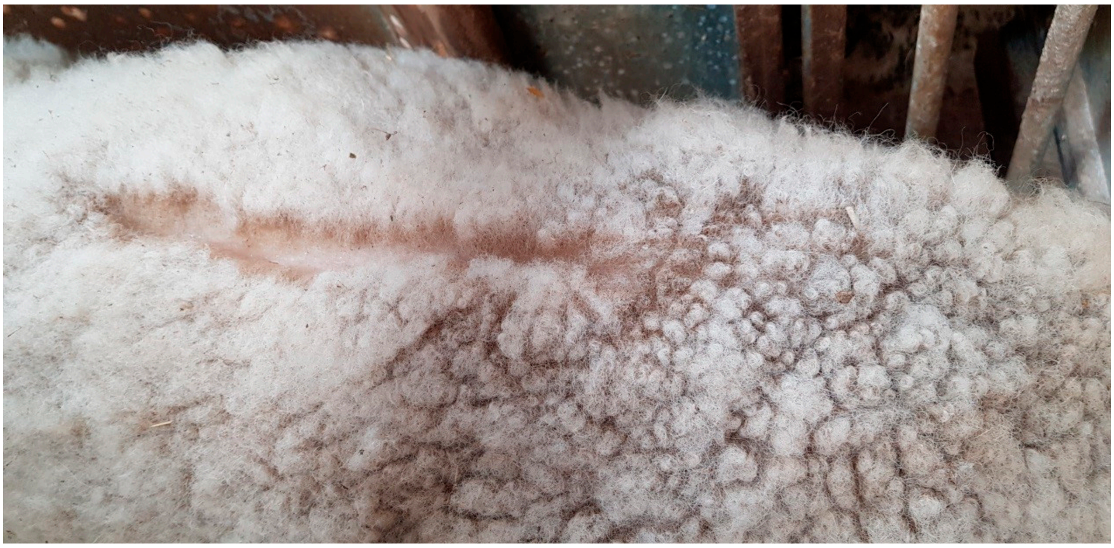

Figure S3. The area of deltamethrin (Deltanil®, Virbac) application post-treatment.
